# Characterization of human islet function in a convection‐driven intravascular bioartificial pancreas

**DOI:** 10.1002/btm2.10444

**Published:** 2022-12-14

**Authors:** Ana G. Santandreu, Parsa Taheri‐Tehrani, Benjamin Feinberg, Alonso Torres, Charles Blaha, Rebecca Shaheen, Jarrett Moyer, Nathan Wright, Gregory L. Szot, William H. Fissell, Shant Vartanian, Andrew Posselt, Shuvo Roy

**Affiliations:** ^1^ Department of Bioengineering and Therapeutic Sciences University of California – San Francisco San Francisco California USA; ^2^ Silicon Kidney LLC San Francisco California USA; ^3^ Department of Surgery University of California – San Francisco San Francisco California USA; ^4^ Division of Nephrology and Hypertension Vanderbilt University Medical Center Nashville Tennessee USA

**Keywords:** convective mass transport, GSIS, intravascular bioartificial pancreas, silicon nanopore membranes, T1D

## Abstract

Clinical islet transplantation for treatment of type 1 diabetes (T1D) is limited by the shortage of pancreas donors and need for lifelong immunosuppressive therapy. A convection‐driven intravascular bioartificial pancreas (iBAP) based on highly permeable, yet immunologically protective, silicon nanopore membranes (SNM) holds promise to sustain islet function without the need for immunosuppressants. Here, we investigate short‐term functionality of encapsulated human islets in an iBAP prototype. Using the finite element method (FEM), we calculated predicted oxygen profiles within islet scaffolds at normalized perifusion rates of 14–200 nl/min/IEQ. The modeling showed the need for minimum in vitro and in vivo islet perifusion rates of 28 and 100 nl/min/IEQ, respectively to support metabolic insulin production requirements in the iBAP. In vitro glucose‐stimulated insulin secretion (GSIS) profiles revealed a first‐phase response time of <15 min and comparable insulin production rates to standard perifusion systems (~10 pg/min/IEQ) for perifusion rates of 100–200 nl/min/IEQ. An intravenous glucose tolerance test (IVGTT), performed at a perifusion rate of 100–170 nl/min/IEQ in a non‐diabetic pig, demonstrated a clinically relevant C‐peptide production rate (1.0–2.8 pg/min/IEQ) with a response time of <5 min.

## 1 INTRODUCTION

Type 1 diabetes (T1D) is an autoimmune disease that affects around 34 million people worldwide.^1^ Clinical islet transplantation by infusion into the portal vein is an attractive treatment for T1D due to its minimally invasive nature. Though islet transplantation has successfully treated patients with unstable T1D,^2^ its wider applicability is hindered by tissue donor shortage and the need for chronic immunosuppressive therapy,^3^ which has been shown to negatively affect the islets and their recipients.^4^ In most cases, achieving insulin independence requires more than one islet infusion, and less than 50% of patients are insulin independent 5 years after intraportal transplantation.^5^

The shortage in donor islets is exacerbated by poor engraftment due to inadequate oxygenation during organ procurement and islet preparation for portal venous infusion. After intraportal infusion and before revascularization, oxygen delivery occurs by diffusion from the surrounding blood and liver tissue, resulting in critically low oxygen tensions, below 40–50 mmHg.^6^ Moreover, a large percentage of islets are destroyed by the instant blood‐mediated inflammatory reaction (IBMIR).^7^ Despite the engraftment challenges and complications from immunosuppressive therapy,^8^, ^9^ islet transplantation remains a promising experimental treatment for T1D because of its ability to reproduce physiologic insulin secretion kinetics and eliminate hypoglycemic episodes.

Encapsulation is a promising approach to transplant islets without systemic immunosuppression. Our group is investigating the development of an intravascular bioartificial pancreas (iBAP) using silicon nanopore membranes (SNM) fabricated using microelectromechanical systems (MEMS) technology.^10^, ^11^, ^12^, ^13^ The SNM feature submicron pores and exhibit high hydraulic permeability at physiologic blood pressures to support increased rates of convective mass transport, and potentially, sustain clinically relevant islet densities by overcoming the limitations of diffusive transport characteristic of extravascular bioartificial pancreas devices.^8^

Previous work has demonstrated that the molecular selectivity and hydraulic permeability of SNM are significantly greater than polymeric membranes.^14^, ^15^, ^16^ Furthermore, studies with murine islets have shown that the SNM serve as an immune barrier under convective mass transport and support insulin production and islet viability both in vitro and in vivo.^10^, ^11^, ^12^ While murine islets are useful for demonstrating preliminary device feasibility, adult human islets are a more clinically appropriate tissue for the iBAP. Hence, we transitioned our testing to adult human islets, and here, we report on the potential of the iBAP to support human islet function. While our previous islet encapsulation studies utilized both silicon nanopore membranes (SNM) with <50 nm‐wide pores^10^ and silicon micropore membranes (SμM) with 1 μm‐wide pores,^12^ we focused this investigation around larger SNM with 450 nm‐wide pores to investigate the effects of higher hydraulic permeability on islet function. First, we modeled in silico the oxygen consumption profiles^17^, ^18^ of islets seeded at three different densities within scaffolds at in vitro (pO2 = 160 mmHg, atmospheric) and in vivo oxygen levels (pO2 = 95 mmHg, arterial). Then, we evaluated their glucose‐insulin kinetic profiles through in vitro glucose‐stimulated insulin secretion (GSIS) assays at various density levels and ultrafiltration rates (normalized to islet quantity, also referred to as “perifusion” rates) of 14–200 nl/min/IEQ. The optimal perifusion rate was determined based on the outcomes of computational modeling and in vitro testing. Finally, a proof‐of‐concept demonstration experiment to evaluate insulin production was conducted in vivo via implantation of an iBAP prototype in a non‐diabetic pig, followed by intravenous glucose tolerance test (IVGTT).

## 2 MATERIALS AND METHODS

### 2.1 Oxygen consumption modeling

#### 2.1.1 Governing equations

The glucose‐dependent oxygen consumption model presented here is an adaptation of the work described by Buchwald.^18^ This model couples convective flow across the microchannels and diffusive transport with consumption rates across the islet tissue. The Navier‐Stokes and mass continuity equations for incompressible Newtonian fluids describe the velocity field (*u*) due to convection (Equations 1 and 2) while the diffusion model is defined by the standard diffusion equation for incompressible fluids (Equation 3):

(1) $$\rho \frac{\partial u}{dt}-\eta \nabla^{2}u+\rho \left(u∙\nabla \right)u+\nabla p=F$$

(2) ∇∙u=0

(3) $$\frac{\partial c}{\partial t}+\nabla ∙\left(-D\nabla c\right)=R-u∙\nabla c$$

where *ρ* denotes density (kg/m^3^), *η* is viscosity (Pa s = kg/m s), *p* corresponds to pressure (Pa = kg/(m s^2^)), and **F** is the volume force (N/m^3^ = kg/(m^2^ s^2^)). In Equation (3), *c* refers to the concentration of the species of interest (mol/m^3^), *D* is the diffusion coefficient (m^2^/s), the *del* operator $\nabla =i\frac{\partial }{\partial x}+j\frac{\partial }{\partial y}+k\frac{\partial }{\partial z}$ and *R* represents the consumption term or the reaction rate (mol/m^3^ s). Both the glucose (Equation 4) and oxygen (Equation 5) consumption rates are assumed to follow Michaelis–Menten‐type kinetics. The metabolic demands of insulin production due to changes in glucose levels affect the local oxygen consumption, which is represented by a modulating function ($\varphi_{o,g}$) dependent on glucose concentration (Equation 6). This function is defined by a base‐rate $\left(\varphi_{\text{base}}\right)$ and a component that changes with metabolic demand along with the insulin secretion rate as a function of glucose concentration. As a first estimate, the base‐rate was assumed to represent 50% of the total rate possible and the scaling factor $\left(\varphi_{\mathrm{sc}}\right)$ was also equal to 1.8. Furthermore, a step‐down function (δ) was also included to account for cell necrosis and suppress the oxygen uptake when its local concentration dropped below the critical value ($C_{\mathrm{cr},\mathrm{oxy}}$).

(4) $$R_{\text{gluc}}=R_{\max,\text{gluc}}∙\frac{c_{\text{gluc}}}{c_{\text{gluc}}+C_{\mathrm{Hf},\text{gluc}}}$$

(5) $$R_{\mathrm{oxy}}=R_{\max,\mathrm{oxy}}∙\frac{c_{\mathrm{oxy}}}{c_{\mathrm{oxy}}+C_{\mathrm{Hf},\mathrm{oxy}}}∙\varphi_{o,g}\left(c_{\text{gluc}}\right)∙\delta \left(c_{\mathrm{oxy}}>C_{\mathrm{cr},\mathrm{oxy}}\right)$$

(6) $$\varphi_{o,g}\left(c_{\text{gluc}}\right)=\varphi_{\mathrm{sc}}\left(\varphi_{\text{base}}+\varphi_{\text{metab}}∙\frac{c_{\text{gluc}}^{n_{\mathrm{ins}\;2,\text{gluc}}}}{c_{\text{gluc}}^{n_{\mathrm{ins}\;2,\text{gluc}}}+C_{\mathrm{Hf},\mathrm{ins}2,\text{gluc}}^{n_{\mathrm{ins}\;2,\text{gluc}}}}\right)$$

The model was developed using a finite element method (FEM)‐based approach implemented with COMSOL Multiphysics (Burlington, MA). Here, δ was COMSOL's smoothed Heaviside function with a continuous first derivative and without overshoot, $\delta \left(c_{\mathrm{oxy}}>C_{\mathrm{cr},\mathrm{oxy}}\right)=\mathrm{flc}1\mathrm{hs}\left(c_{\mathrm{oxy}}-1.0\times 10^{-4},0.5\times 10^{-4}\right)$. The parameters selected for the generalized Michaelis–Menten expression remained the same for each species (see Reference 15 and Table 1). All islets were assumed to be the size of an islet equivalent (1 IEQ = 150 μm in diameter) and a stepwise increase to 28 mM glucose concentration^19^ was added to correlate the in vitro insulin production data to the spatial oxygen distribution at any given time in the GSIS assay.

**TABLE 1:** Summary of islet perfusion rates within the iBAP

IEQ (No.)	Islet density (v/v%)	Ultrafiltration rate (μl/min)	Perifusion rate (nl/min/IEQ)
3600	20.0	50	14
1800	10.0	50	28
1800	10.0	100	56
500	2.5	50	100
500	2.5	100	200

#### 2.1.2 Model geometry and boundary conditions

**FIGURE 1:**
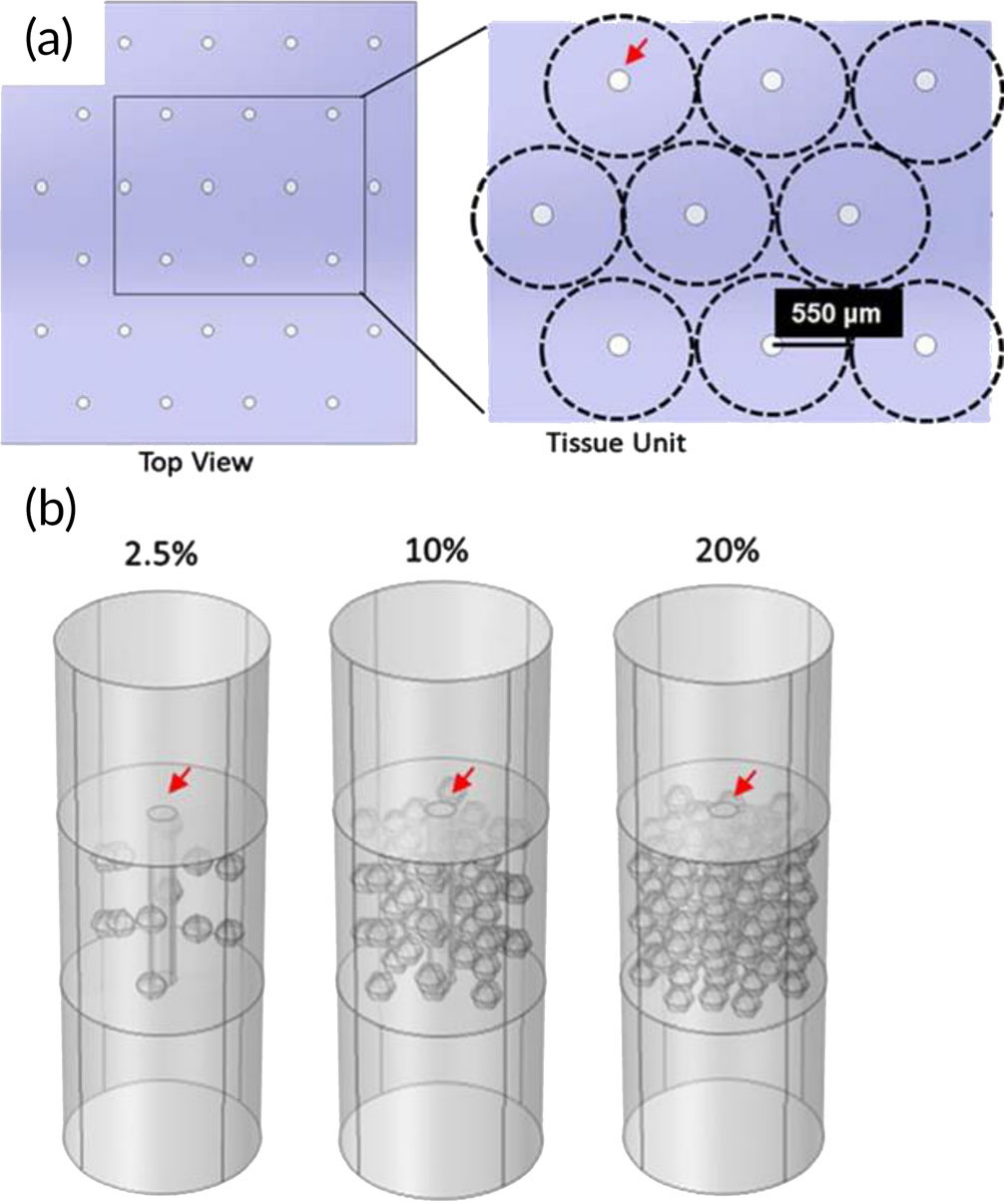
Oxygen model setup of islet scaffold (a). Top view of the islet scaffold design with hexagonal arrangement of 150‐μm diameter microchannels (red arrows) and a 550 μm radius tissue unit (dashed outline). (b) 3‐D geometries of the tissue unit (3 mm‐height, 150 μm diameter) showing islet distributions at 2.5, 10.0, and 20.0 v/v% loading densities.

Figure 1a shows a schematic of the hexagonal arrangement in our microchannel islet scaffold design displaying a 1100 μm distance from center to center between microchannels. Single microchannel models of the 2.5%, 10.0%, and 20.0% (v/v) islet densities shown in Figure 1b were created in COMSOL and their outer walls were defined by the symmetry boundary condition. Comparisons between iterations of extra‐fine and normal element size mesh showed no major differences in the results. Therefore, all simulations presented here were ran using COMSOL's default normal element size mesh. For all liquid–solid interfaces, the no‐slip (**u** = 0 m/s) boundary condition was used. Obeying Henry's Law, the inflow or initial concentrations of oxygen were set to 0.1284 mol/m^3^ (95 mmHg) and 0.2156 mol/m^3^ (160 mmHg) for in vivo or in vitro concentrations, respectively. The basal glucose concentration (**L1**) was set at 5 mM. In addition, convective flow was employed to solve for outflow of species **n** (−*D*_*i*_∇*c*_*i*_ = 0), and outlet pressure at the outlet was assumed to be 0 Pa with “no viscous stress.” The continuity equation was used to solve for the diluted species across the islets. Table 1 summarizes the perifusion rates tested for the various islet density levels and ultrafiltration rates.

### 2.2 Human islet receipt and culture

Freshly isolated human islets were extracted from deceased donor pancreata by the UCSF Islet Production Core (San Francisco, CA). The islets were cultured overnight after isolation at 5% CO2 and 37°C in Connaught Medical Research Laboratories (CMRL) 1066 medium (Nucleus Biologics, CA) NIH CIT supplemented with the addition of 0.5% Human Serum Albumin (Grifols, Spain), 10 U/mL of Heparin (Fresenius Kabi, Germany), 2 μg/mL DNAse (Genentech, CA) and 20 μg/mL Ciprofloxacin (Hospira, IL). Human islets were also sourced from Prodo Laboratories, Inc (Aliso Viejo, CA) and cultured with their proprietary medium (PIM(S) supplemented with Human AB Serum).

### 2.3 Glucose‐stimulated insulin secretion

**FIGURE 2:**
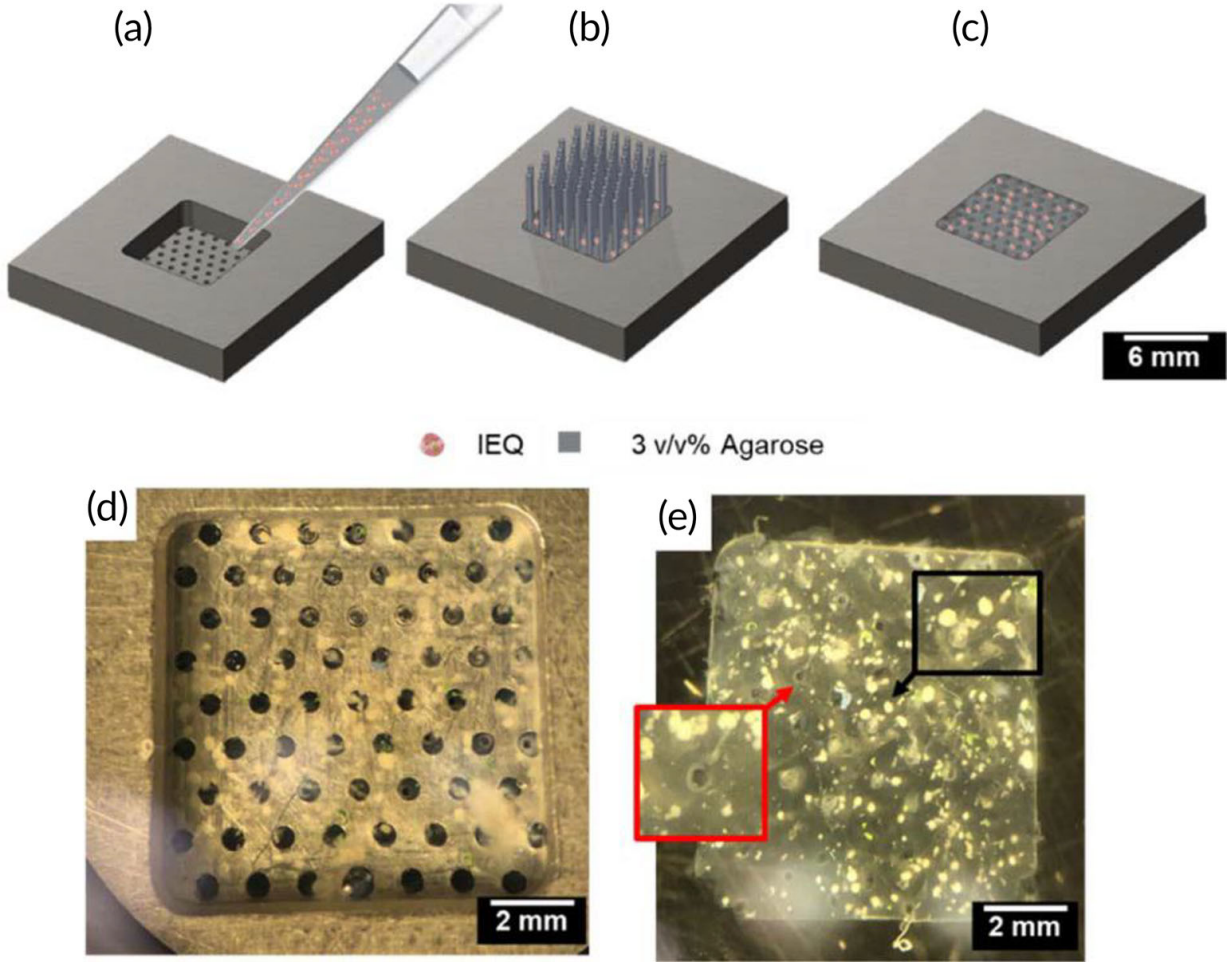
Islet scaffold fabrication and chamber design (a). Islet scaffold construction on the 316 L stainless‐steel chamber consisting of a hexagonal configuration of 56 holes (~150 μm‐diameter). (b) Wire‐array alignment with islet chamber for islet scaffold construction and (c) isometric view of islet scaffold showing hexagonally arranged microchannels and islets. Encapsulated human islets in (d) the islet chamber and (e) islet scaffold with microchannels removed from the chamber (post‐testing in the iBAP). The red sectioned arrow encloses the 150 μm microchannels with radial diffusion distances ≤400 μm and the black arrow shows the IEQs.

The islet scaffold presented here builds on the work reported by Song et al.^10^ The chambers were fabricated from biocompatible 316L stainless‐steel grade metal that was CNC machined by Hayes Manufacturing Services, Inc. (Sunnyvale, CA). Approximately 2.5%, 10.0%, and 20.0% (v/v) of islet equivalents (1 IEQ = 150 μm diameter islet) per 36 μl chamber were immobilized in 3% (w/v) ultra‐low gelling agarose (Sigma: 9012‐36‐6) scaffolds. Islets were mixed with the agarose solution at 37°C and pipetted into the void region above the hexagonal arrangement (Figure 2a). Next, an array of wires was aligned with the islet chamber and pushed through to create ~150 μm‐diameter microchannels while curing at 4°C for 10 min (Figure 2b). After curing the islet‐agarose mixture in the islet chamber and removing the wire‐array, islet scaffolds exhibited microchannels with ~800 μm center‐to‐center separation (Figure 2c). This inter‐microchannel distance, which resulted from constraints of the available fabrication methods, is lower than the tissue unit in the oxygen model, which can therefore be considered a “worst‐case” scenario. Figure 2d and e shows representative images of the islet scaffold in the chamber at 2.5% (v/v) density, which corresponds to the scaffolds tested at either 100 or 200 nl/min/IEQ (see Table 1).

The mock circuit loop was set up as previously described^10^, ^11^, ^12^ and all connections were made with platinum‐cured silicone LS‐25 tubing (Cole Parmer: 96410‐14). d‐Glucose (Sigma‐Aldrich: SLBX5177) was added to the basal 5 mM media supplemented with 10% fetal bovine serum (Gibco: 16000‐04) to create a high glucose concentration level of 28 mM. A stabilization period was implemented with 5 mM glucose for 2 h and then ultrafiltrate samples were collected for 16 min (**L1**). The concentration was subsequently increased to 28 mM (**H**) for 30 min and then reduced to 5 mM for 32 min (**L2**). The insulin content in the ultrafiltrate samples was quantified using an enzyme‐linked immunosorbent assay (ELISA) kit (Mercodia: 10‐1113‐01; Uppsala, Sweden). Absorbance values were acquired at a 450 nm wavelength using a SpectraMax M5 microplate reader (Molecular Devices: MV06103; Washington, DC). To calculate the insulin production relative to the perifusion rate, the insulin concentration was multiplied by the ultrafiltration rate and then divided by the number of IEQs within the islet scaffold. The stimulation index was calculated as the ratio of the insulin production at the first peak during high glucose exposure to the baseline average insulin production at low glucose.

### 2.4 In vivo assessment

#### 2.4.1 iBAP device assembly and preparation

**FIGURE 3:**
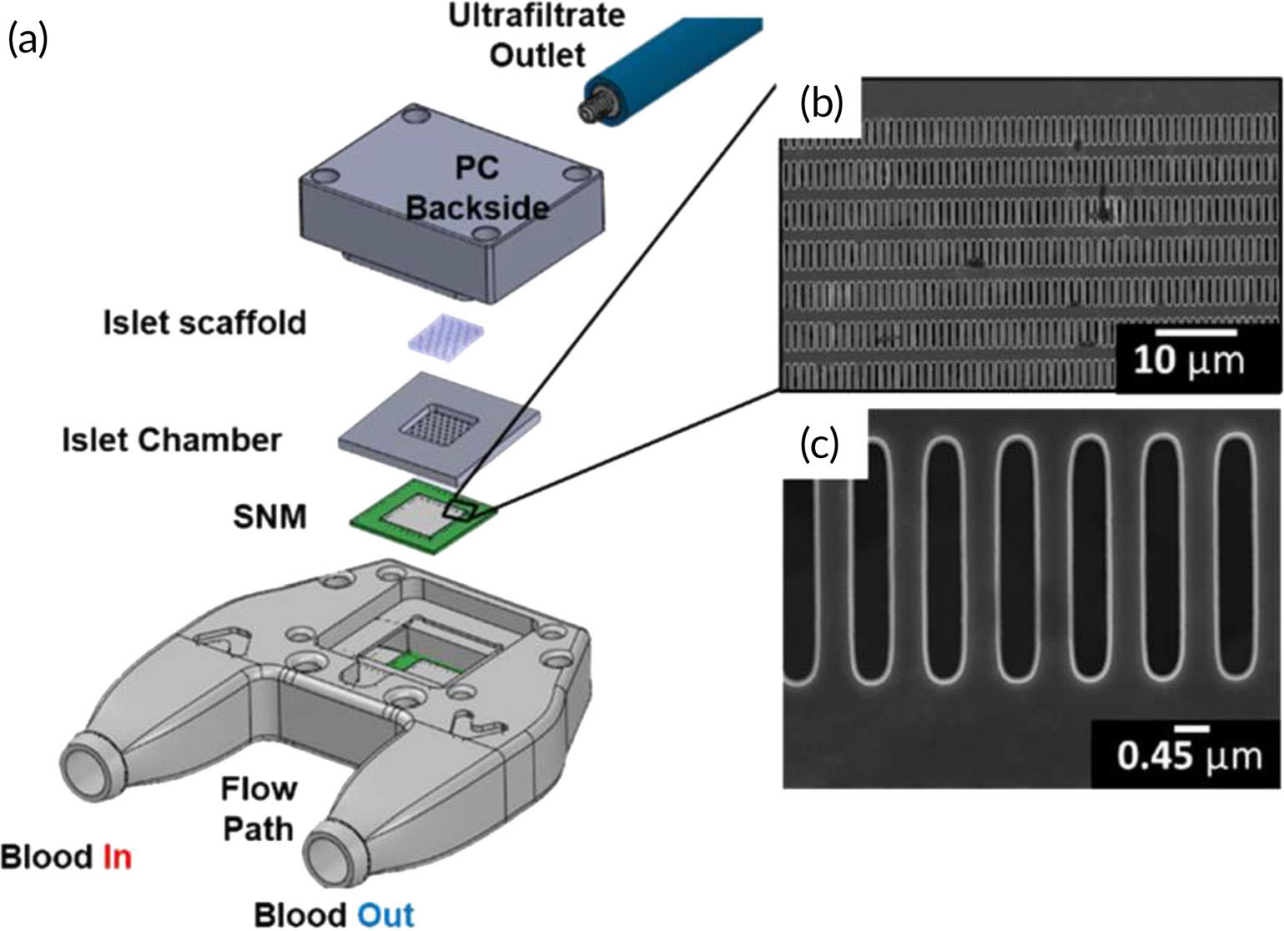
Assembly of the SNM‐based iBAP (a). Exploded view of the iBAP components with the islet chamber design exhibiting a 6 × 6 × 1 mm cavity (36 μl volume). (b) Scanning electron microscopy (SEM) image of the SNM (1.8k magnification). (c) Close‐up SEM image of the 450 nm‐wide pores (16k magnification).

The SNM‐based iBAP (Figure 3a) was comprised of a polycarbonate blood flow‐path sandwiched by an SNM‐islet chamber stack on each side and sealed with polycarbonate backsides containing ultrafiltrate (UF) outlets. SNM were microfabricated as previously described.^20^ The patterned silicon wafer was diced into 1 × 1 cm squares resulting in single chip SNM with an active membrane area of 36 mm^2^ and 3.12 × 10^6^ pores per chip. Scanning electron microscopy of the SNM showed uniform pores with 450 nm width, 4 μm length and 1 μm in height (Figure 3b and c). To prevent protein fouling, the SNM surface was coated with diethylene glycol dimethyl ether, which is also known as diglyme, (Sigma:111‐96‐6) at Plasmatreat USA, Inc. (Hayward, CA). This step was achieved by performing a two‐stage treatment in the Plasmatreat Aurora™ plasma reactor with dual side‐wall electrodes. The plasma treatment step consisted of an oxygen plasma cleaning at a flowrate of 250 cm^3^/min, for 2 min followed by polyethylene oxide plasma polymerization for 20 min at 300 W with argon at 6 cm^3^/min. The chamber was evacuated to 25 mTorr at the beginning and end of each stage. Ellipsometry measurements after vapor diglyme deposition revealed an average coating thickness of 15 nm. All polycarbonate components and SNM were disinfected with 70% ethanol for 45 min and washed with sterile water inside a laminar flow hood three times. The stainless‐steel islet chambers were steam autoclaved at 121°C along with the fastening screws and the assembly tools, while the agarose solution was sterile filtered and kept at 37°C during the islet scaffold construction. The device was assembled in a sterile field within the laminar flow hood to house 500 IEQ (2.5%, v/v) in the islet chamber and primed with CMRL 1066 medium for transportation to the animal surgical suite.

#### 2.4.2 Intravenous glucose tolerance test

**FIGURE 4:**
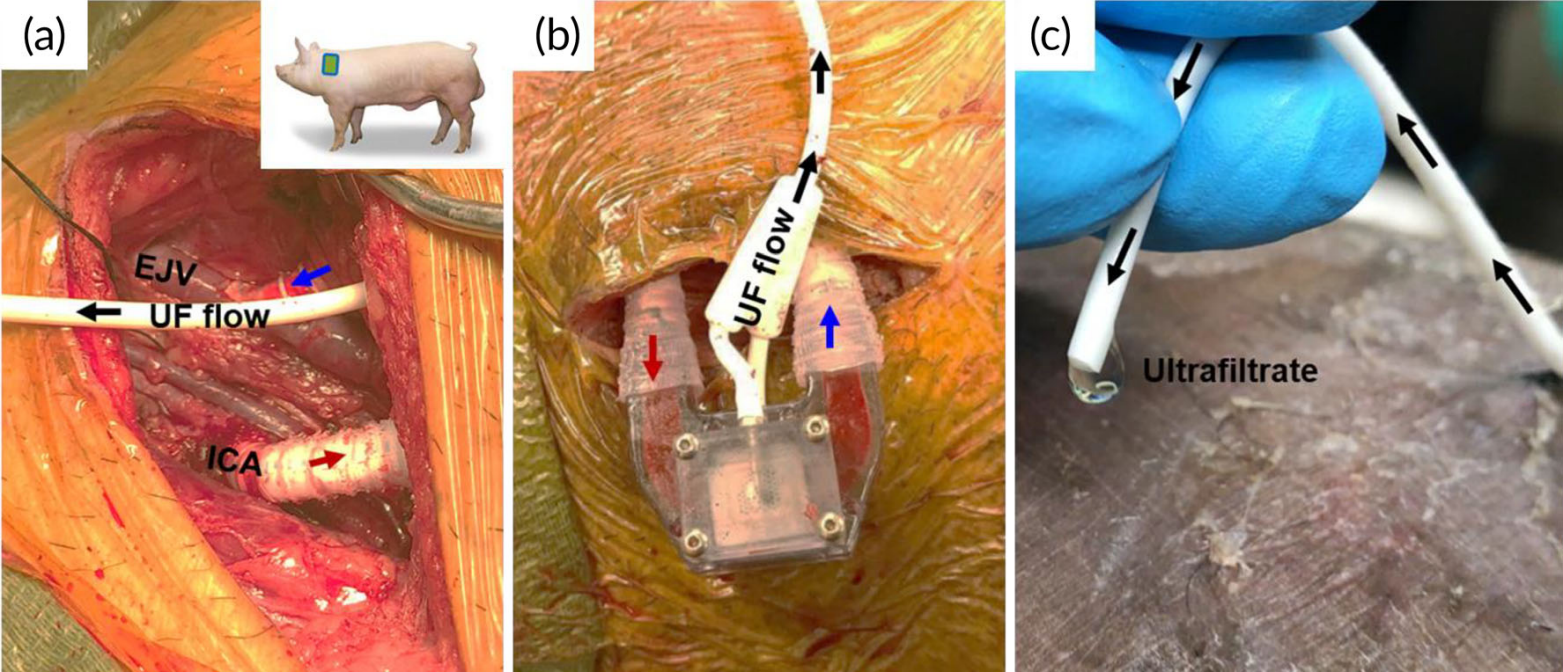
In vivo testing of the SNM‐based iBAP (a). Anatomic location for device placement in pigs (inset) showing vascular anastomoses of the iBAP to the internal carotid artery (ICA) and the external jugular vein (EJV). (b) iBAP with blood flow where the red and blue arrows denote the inlet arterial and venous flows, respectively. (c) Clear ultrafiltrate (UF) at tip of catheter connected to the exit of the iBAP islet chamber.

The IVGTT study with the iBAP in a single 38‐kg non‐diabetic Yucatan pig was approved by the Institutional Animal Care and Use Committee (IACUC) review committee at Covance (San Carlos, CA). The animal was treated with 325 mg aspirin daily for 3 days and fasted overnight prior to surgery. The animal was placed under inhaled general anesthesia, and monitored continuously visually and using telemetry, pulse oximetry, and an esophageal temperature probe during all procedures. Following surgical dissection of the right anterolateral neck, thin walled 6 mm diameter polytetrafluoroethylene (ePTFE) vascular grafts (Gore‐Tex SRRT06030040L) were anastomosed to the carotid artery and external jugular vein and then connected to the flow inlet and outlet of the iBAP device (Figure 4a). Prior to vessel clamping and anastomosis creation, the animal was anticoagulated with intravenous heparin 100 u/kg, which was readministered to maintain activated clotting time between 250 and 350 s. The graft‐device interface was reinforced with an injection molded Dragon Skin 10a silicone coating (Smooth‐on, Inc., Pennsylvania) applied circumferentially onto the exterior of ePTFE graft. A 7 Fr silicone Hickman catheter (Becton Dickinson 0600310) (Figure 4b) was connected to stainless steel barbs for ultrafiltrate collection (Figure 4c). While perifused, and with the pig under general anesthesia, the encapsulated islets were stabilized for 75 min at fasting blood glucose levels of 80–100 mg/dl. The pig was then subjected to an intravenous glucose tolerance test (IVGTT) wherein 0.5 g of d‐glucose/kg body weight was administered as a bolus via a central line. Ultrafiltrate samples were collected at time zero and then every 4–15‐min intervals for 90 min. Following completion of sample collection, the animal was euthanized according to AVMA Guidelines for Euthanization of Animals.^21^

The IVGTT protocol employed here is an adaptation of the work by Hara et al.^22^ Blood glucose was measured at every timepoint with an Accu‐Chek Compact Plus glucometer. Due to the high cross reactivity between human and porcine insulin, a human C‐peptide ELISA (Mercodia: 10‐1141‐01) was used to test the ultrafiltrate samples to determine contribution of the encapsulated islets.

### 2.5 Statistical analysis

Results were expressed as the mean ± standard deviation of the mean (SD). Multiple sample comparisons were done with two‐way analysis of variance (ANOVA) followed by post hoc Tukey test while sample pairs were evaluated using Student's *t*‐test with the Holm‐Sidak correction method. All statistical analyses were performed with GraphPad Prism 6 (San Diego, CA) and *p* values <0.05 were considered statistically significant.

## 3 RESULTS

### 3.1 Microchannel oxygen modeling

**FIGURE 5:**
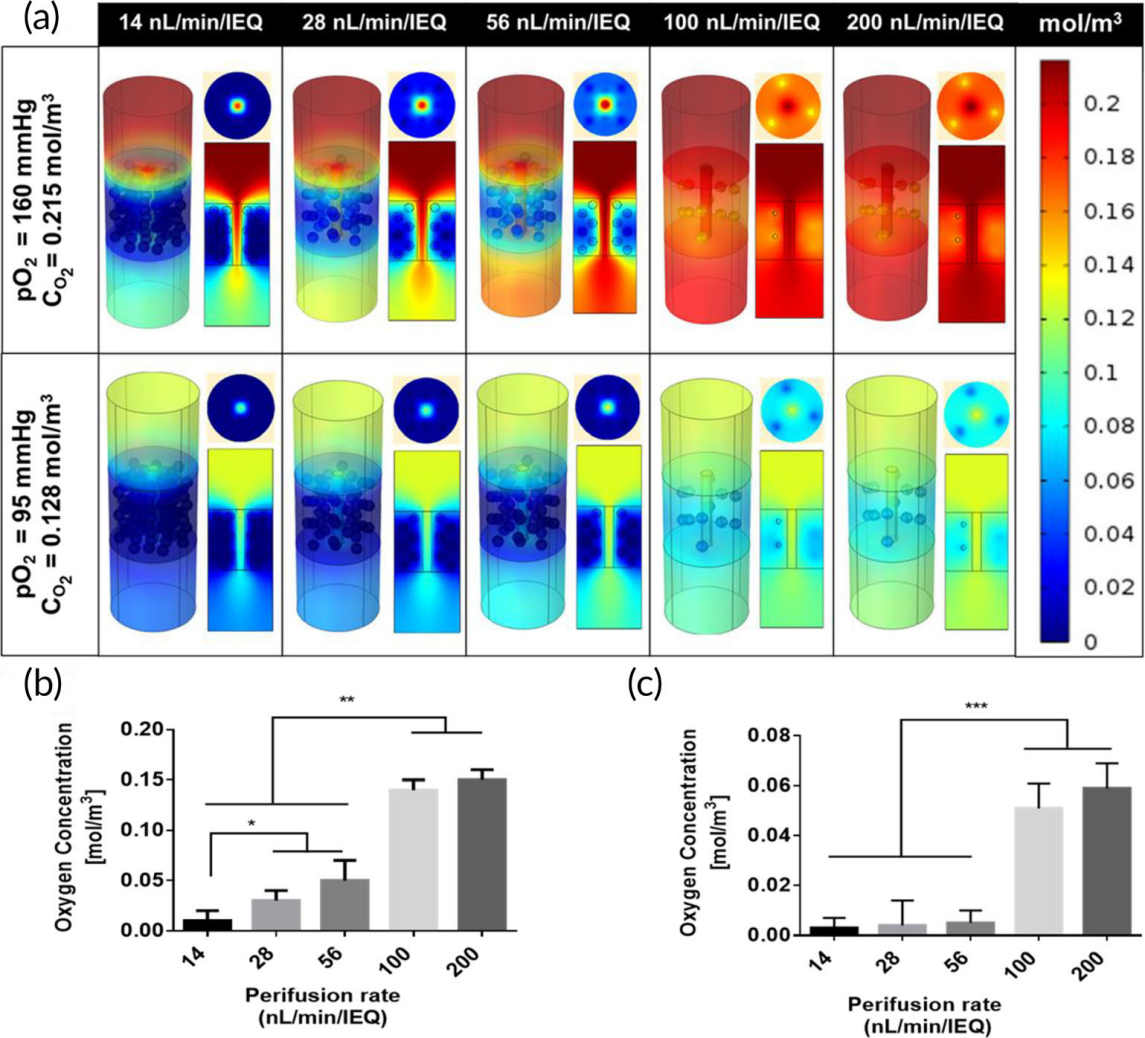
Simulated in vitro and in vivo oxygen concentrations in the tissue unit as a function of islet perifusion rate 10 min after introducing glucose (28 mM) in the bulk fluid (a). Surface plots of oxygen concentration gradient across the tissue unit with radial and longitudinal cross‐sections halfway through the unit. The average oxygen concentration at the islet cores farthest from the microchannel (worst‐case scenario) plotted at (b) 160 mmHg and (c) 95 mmHg inlet pO_2_. Simulations at arterial pO_2_ levels suggest that at least 100 nl/min/IEQ (***) is required to be within the oxygen threshold of uninhibited maximal insulin production (0.034 mol/m^3^). See Table 1 for correlation between perifusion rate and islet density loading levels. Statistical significance is expressed as *p < .05 and **p < .001

The perifusion rates were modeled at incoming atmospheric (160 mmHg) and arterial (95 mmHg) oxygen tensions to recreate in vitro and in vivo conditions (Figure 5a). The resulting oxygen profiles were extracted 13 min after the introduction of high glucose to capture the maximal first‐phase insulin release. The oxygen concentration steadily decreased with the radial distance of the tissue unit as oxygen diffused from the microchannel into the agarose‐islet region where it was consumed by the islets. As expected, the islets farthest away from the microchannel in the radial direction showed the lowest oxygen concentration and represented the worst‐case scenario within the islet scaffold. Also, the oxygen concentration within the tissue unit (both axial and radial directions) increased as the incoming perifusion rate was increased. The post‐processing cut‐line feature in COMSOL was used to visualize the oxygen concentration in the worst‐case scenario for each condition. The results obtained from the simulation at atmospheric oxygen tension indicate that 28 nl/min/IEQ (10.0% density, 50 μl/min) is the lowest tested rate supporting islet function; since its worst‐case scenario drops slightly below 25 mmHg (0.034 mol/m^3^), which corresponds to the threshold for uninhibited maximal insulin production^23^, ^24^ (Figure 5b). Simulations at arterial pO2 levels revealed that the maximal insulin production was supported by perifusion rates ≥100 nl/min/IEQ (2.5% density, 50 μl/min) (Figure 5c).

### 3.2 Effect of perifusion rate in vitro, pO_2_ = 160 mmHg

**FIGURE 6:**
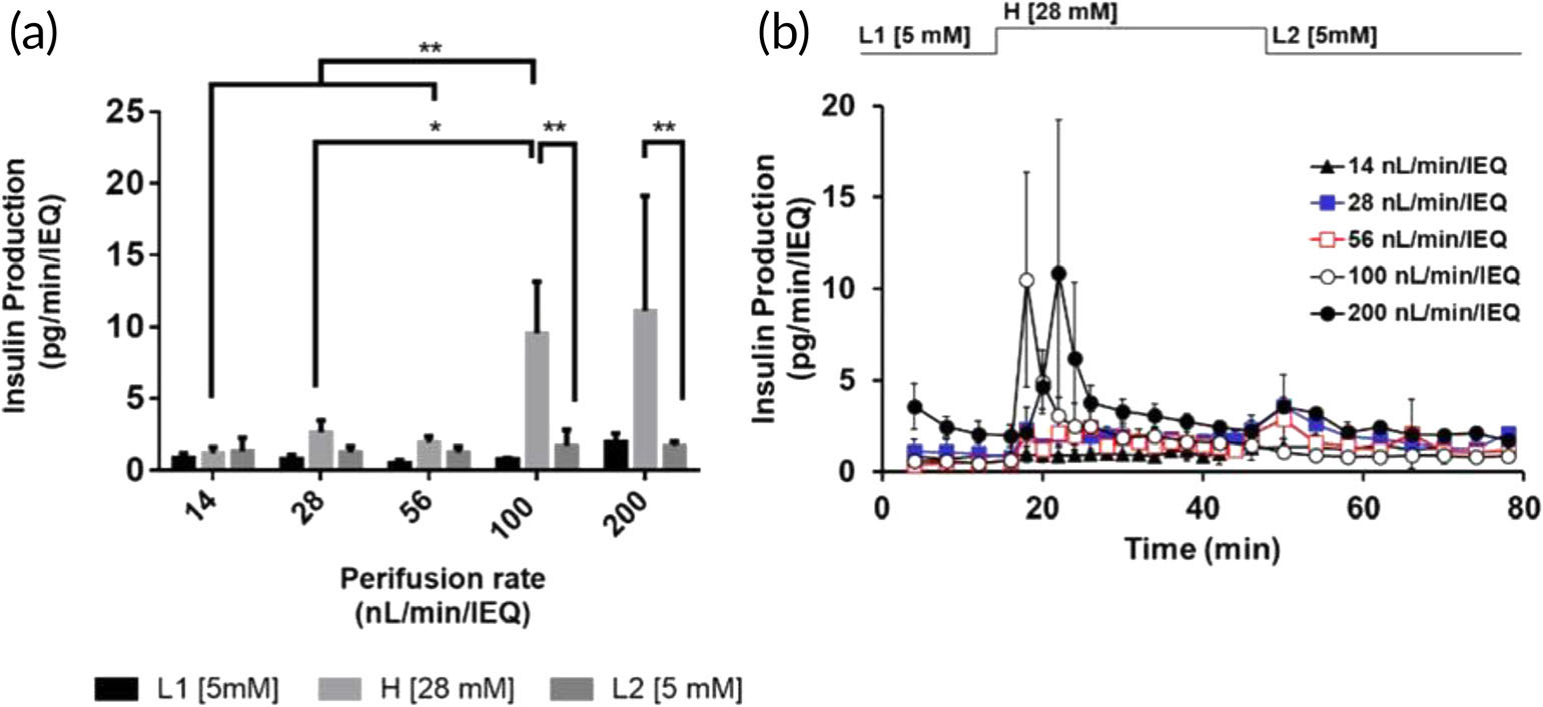
Glucose‐stimulated insulin secretion (GSIS) at 160 mmHg pO2 (a). Average pre‐ and post‐stimulatory phases (L1 and L2) and phase 1 of the physiologic biphasic response. (b) GSIS curves for 14, 28, 56, 100 and 200 nl/min/IEQ over time. A biphasic response was observed for a minimum perifusion rate of 28 nl/min/IEQ. Higher perifusion rates resulted in higher stimulation and insulin production (*) as well as greater shutdowns in insulin production (**, H → L2). See Table 1 for correlation between perifusion rate and islet density loading levels. Statistical significance is expressed as *p < .05 and **p < .001

The five perifusion rates that were modeled were also evaluated for GSIS. In vitro results show that 28 nl/min/IEQ (10.0% density, 50 μl/min) is the lowest perifusion rate that can sustain islet function in the microchannel islet scaffold as a marked response to changes in glucose levels can still be observed (Figure 6a). Conversely, the 14 nl/min/IEQ rate (20.0% density, 50 μl/min) demonstrated poor response to glucose stimulation. A 2‐way ANOVA analysis with post hoc Tukey test corroborates this claim as there was no statistical significance upon comparing the L1, H and L2 phases for the lowest perifusion rate tested, whereas insulin secretion at high‐glucose (H) was consistently higher than at low glucose (L1) for all others. In general, higher insulin production is achieved at higher perifusion rates. The 100 nl/min/IEQ rate (2.5% density, 50 μl/min) exhibited greater insulin production at the 28 mM glucose level compared to lower perifusion rates and showed no statistical significance compared to the insulin production rate corresponding to 200 nl/min/IEQ (2.5% density, 100 μl/min) at the same glucose level. Interestingly, the 100 nl/min/IEQ rate resulted in a higher stimulation index compared to that corresponding to the 200 nl/min/IEQ rate; these indices were 13.86 ± 7.29 and 5.30 ± 2.48, respectively. Additionally, the insulin production decreased significantly when transitioning from the stimulatory to post‐stimulatory phases (H → L2) for the two highest perifusion rates studied (Figure 6b).

### 3.3 In vivo intravenous glucose tolerance test

**FIGURE 7:**
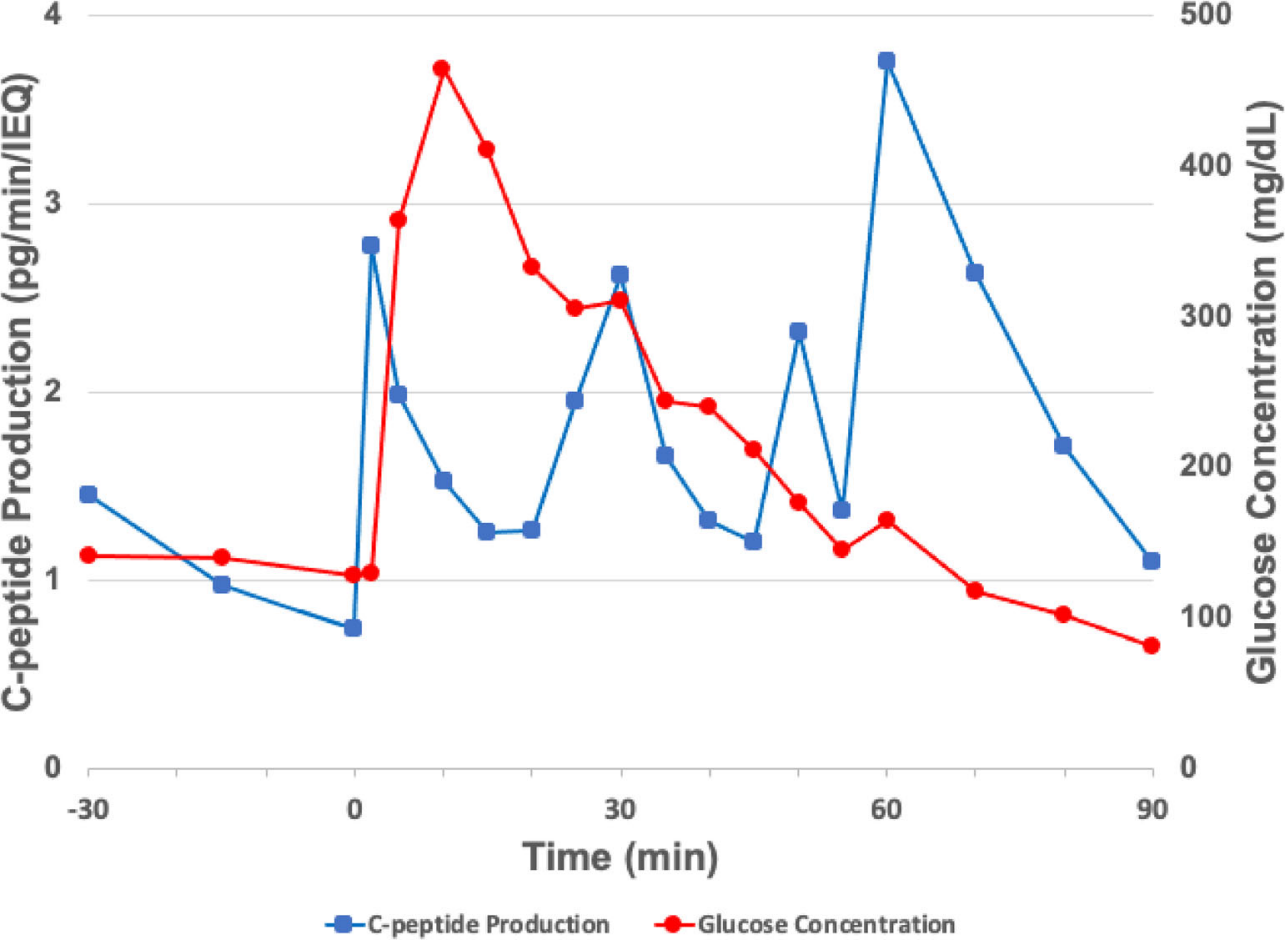
In vivo intravenous glucose tolerance test (IVGTT) in a non‐diabetic pig. The SNM‐based iBAP with 500 IEQ and a perifusion rate of 100–170 nl/min/IEQ showed stable C‐peptide production in the fasting period. Time 0 marks the administration of the glucose bolus, and elevated C‐peptide production is observed as the glucose concentration increases. The C‐peptide production approached its basal level as the blood glucose returned to fasting levels.

In vivo results were assessed based on the human C‐peptide concentration in the ultrafiltrate using a pig model with an implanted device as described in the Methods. With an islet density of 2.5% (v/v), the implanted iBAP exhibited ultrafiltration of 50–85 μl/min through the microchannels, which resulted in perifusion rates of 100–170 nl/min/IEQ. It should be noted that the ultrafiltrate generated via the SNM and exposed to the islets is free of hemoglobin. These perifusion rates led to a stable (but slightly downward trending) C‐peptide production during exposure to fasting blood glucose (BG) levels in the animal (Figure 7). Since the observed perifusion rates in vivo remained between 100 and 200 nl/min/IEQ, the insulin/C‐peptide secretion can be correlated to the predicted oxygen profiles at arterial pO2 levels. The pre‐stimulatory phase, which served as a stabilization period, of the IVGTT established C‐peptide baseline of 1.06 ± 0.32 pg/min/IEQ. After administration of the glucose bolus, elevated C‐peptide production was observed immediately thereafter (2.77 ± 0.05 pg/min/IEQ), and at 30, 50 and 60 min of the IVGTT (2.62 ± 0.21, 2.31 ± 0.08, and 3.76 ± 0.01 pg/min/IEQ, respectively) (Figure 7). The C‐peptide production oscillated over time with a post‐glucose bolus average of ~2 pg/min/IEQ. As the BG levels decreased, the C‐peptide production approached its pre‐stimulatory, or stabilization, value of ~1 pg/min/IEQ.

## 4 DISCUSSION

Advances in islet encapsulation have led to the creation of both extravascular and intravascular bioartificial pancreas (BAP) devices,^25^ with significantly more effort focused on extravascular devices. Extravascular devices rely on passive diffusion of solutes, with diffusion distances typically greater than 500 μm.^26^ The oxygen tension at the outer surface of extravascular devices is at most 45 mmHg,^27^ which, coupled with the large diffusion distance, may result in poor islet oxygenation, necrosis, and limited and delayed glucose‐stimulated insulin secretion (GSIS).^28^ However, oxygenation in extravascular BAP devices can be enhanced by implementing oxygen‐generating materials in the islet microenvironment,^29^, ^30^ attaching an exogenous oxygen supply,^31^, ^32^, ^33^ or incorporating an in situ oxygen generating device.^34^ Recently, Yang et al. reported a convection‐enhanced macroencapsulation device (ceMED), which can be categorized as an extravascular BAP that delivers insulin through diffusion into subcutaneous tissue.^35^ Another promising approach is the use of pre‐vascularized devices to facilitate revascularization in the islets.^36^, ^37^

Intravascular BAP devices may improve GSIS compared to extravascular devices as they can deliver arterial pO2 levels (80–100 mmHg) to the encapsulated islets, compared to the low pO2 levels (10–50 mmHg) at the surface of extravascular devices.^38^ Previous groups have achieved long‐term xenogeneic islet function in intravascular diffusion‐based devices without immunosuppression^39^, ^40^, ^41^; however, the need for exogenous insulin was not fully eliminated and their translation to the clinic was obstructed by device patency issues and failure at the artery/device connection.^42^ Furthermore, GSIS delays were observed in both the diffusion‐based^43^ and, to a lesser extent, in the convection‐based intravascular devices^44^ previously investigated. The low hydraulic permeability of polymer membranes in these devices limited the mass transport of oxygen and likely led to deficient GSIS outcomes.^45^

Safety concerns around complications with prior intravascular BAP devices have limited enthusiasm for clinical adoption, and consequently, recent research efforts are largely focused on extravascular devices. However, the advances in biomaterials and minimally invasive surgical techniques over the last two decades offer some hope for the successful development and clinical translation of safe and effective intravascular BAP devices. These innovations along with the experience in the surgical implantation, hemodynamic changes, and complication management of over 75,000 prosthetic grafts in hemodialysis patients^46^ have generated a knowledge database that can be used for the clinical implementation of next‐generation intravascular devices for islet therapy. Indeed, intravascular BAP devices are still under investigation as demonstrated by the recent report by Han et al., where acellular arteriovenous grafts are embedded islets on the outer surface.^47^

In the current work, we used a computational model of oxygen consumption to analyze a scaffold unit with 1100 μm center‐to‐center spacing between microchannels, while the fabricated scaffolds exhibited a center‐to‐center separation of 800 μm. The shorter diffusion distance in the fabricated scaffolds should result in better oxygenation for most islets than those shown for computational model (Figure 5b and c), which is equivalent to a “worst‐case” scenario. The in vitro results demonstrated that the scaffold geometry in the iBAP produced comparable outcomes to traditional islet perfusion systems^48^; the GSIS curves above 14 nl/min/IEQ exhibit all the features associated with healthy, non‐encapsulated human islets as reported by Henquin and co‐workers.^49^ These features include a biphasic secretion pattern whose first phase is indicated by a peak in insulin production within the first 15 min of high glucose exposure followed by a sustained second phase, and a transient increase after switching back to low (5 mM) glucose exposure. This transient increase in insulin production is followed by a drop to baseline insulin production at 5 mM. While the dampening in GSIS response can be attributed to the insufficient oxygen supply at <28 nl/min/IEQ, high perifusion rates can sometimes adversely affect the glucose‐insulin kinetics profile, possibly due to shear‐induced damage at the periphery of the islets.^50^ This effect might indeed be the case for the 200 nl/min/IEQ perifusion rate, where a slight delay in insulin production and a lower stimulation index were observed, the latter which is due to higher baseline insulin production at low glucose. Despite the slight delay in the GSIS profile, the 200 nl/min/IEQ perifusion rate still exhibits the desirable features of the biphasic response.

After connection to the vasculature of the non‐diabetic swine, the SNM‐encapsulated human islets, which are exposed to hemoglobin‐free ultrafiltrate, exhibited promising C‐peptide production. The C‐peptide production curve, which peaked at ~4.0 pg/min/IEQ, generated from the in vivo IVGTT with the non‐diabetic pig corresponds to in vitro perifusion rates between 100 and 200 nl/min/IEQ. During the stabilization period at fasting glucose levels, the C‐peptide production was stable but trending slightly downward and comparable to in vitro studies at low glucose levels. This data suggests that human islets within the iBAP can sense plasma blood glucose and secrete insulin in a physiologically normal pattern during fasting glucose levels.^48^ Furthermore, after initiating the IVGTT, the adult human islets rapidly released insulin, as determined by human C‐peptide measurements, with a time delay of <5 min. This rapid release of insulin is a key requirement for a functional bioartificial pancreas to achieve normoglycemia. It has been estimated the GSIS response must be <15 min for normal physiologic BAP funciton.^51^ Interestingly, the glucose‐insulin kinetics exhibited an oscillating profile as opposed to the biphasic pattern observed during in vitro GSIS studies with the SNM‐based iBAP and islet perfusion studies with adult human islets.^49^, ^52^, ^53^ Instead, the islets displayed a pulsatile secretion pattern (Figure 7), with decreasing periodicity or faster oscillations as the BG approached a basal value. This oscillation phenomenon has been reported from plasma insulin concentrations in post‐absorptive periods.^54^ It is possible that the C‐peptide measurements and insulin response from the human islets may have been affected by the function of the native porcine pancreas. The final spike in C‐peptide production at the end of the IVGTT could be due to the slight increase in BG, which is known to increase the amplitude of oscillations in insulin secretion, or simply within measurement error of the glucometer.

Our investigation is associated with several limitations that must be satisfactorily addressed for the successful development of a scaled‐up iBAP suitable for future clinical translation. The in vitro studies were short‐term and conducted with an iBAP prototype that held no more than 3600 islets. While the in vivo pig study with the implanted iBAP showed some promising preliminary data, it was performed with lowest islet density (2.5%) for just 90 min and with a single pig. Future work will need to examine effects of increased islet loading density levels and C‐peptide trends during the low glucose phases of the IVGTT. Studies will need to be conducted with a statistically significant number of pigs and for longer periods (few hours to multiple days to many months). To compare our iBAP results more readily with published literature on encapsulated islets, experiments could be performed at low and high glucose levels of 2.8 and 16.7 mM, respectively, and use a physiologic salt solution as the medium for GSIS experiments to avoid the confounding influence of insulinotropic factors in culture media. For translational relevance to the clinical setting, the iBAP design will need to be scaled up to house an increased SNM area, and therefore generate higher ultrafiltrate volumes, to support a greater number of islets.

## 5 CONCLUSIONS

The prototype SNM‐based iBAP supported adult human islet function in vitro as well as in a healthy Yucatan pig. The oxygen profile models showed that a minimum perifusion rate of 28 nl/min/IEQ and 100 nl/min/IEQ is needed to sustain islets for glucose production in vitro and in vivo, respectively. The animal test demonstrated the potential feasibility of a future scaled‐up device to provide clinically relevant C‐peptide production with 100–200 nl/min/IEQ perifusion rates. Based on simulated oxygen profiles and insulin production in both basal and stimulatory phases, the results of this investigation will inform future islet dosing and device scalability studies required to systemically deliver insulin to treat pigs with chemically induced T1D, and ultimately, T1D patients.

## Data Availability

The data that support the findings of this study are available from the corresponding author upon reasonable request.
